# P-365. Outbreak of Carbapenemase-Producing Organisms in the Cardiovascular ICU Linked to "Hoppers"

**DOI:** 10.1093/ofid/ofae631.566

**Published:** 2025-01-29

**Authors:** Jordana H Fersovich, Maureen Buchanan-Chell, Stephanie W Smith

**Affiliations:** University of Alberta, Edmonton, Alberta, Canada; Alberta Health Services, Edmonton, Alberta, Canada; University of Alberta, Edmonton, Alberta, Canada

## Abstract

**Background:**

Between June and October 2023, a Canadian cardiac centre recognized a growing number of carbapenemase producing organisms (CPO) in patients in the cardiovascular intensive care unit (CVICU). The organisms were mainly KPC-producing Enterobacterales. Following declaration of an outbreak in the CVICU at the Mazankowski Heart Institute, we investigated the sources of CPO across the unit and in patient rooms to determine environmental reservoirs and mechanisms of patient transmission.

**Methods:**

We employed prevalence screening to identify cases, environmental sampling to identify source, and comparison of patient strains utilizing whole genome sequencing (WGS) during the investigation.

**Results:**

Between June and October 2023, five patients were identified as being colonized or infected with CPO-KPC. WGS of patient samples confirmed clonality with less than 10 SNV differences in the plasmid . Environmental sampling revealed drains in the hand-hygiene sinks and medical sinks used for disposal of liquid clinical waste (“hoppers”) harboured CPO. Using GlowGerm^TM^, we found that the faucets of the hoppers resulted in a ∼30cm splash zone causing contamination of medical supplies and health care workers. GlowGerm^TM^ did not reveal splash from drains of hand hygiene sinks. Because the hoppers were the only temperature controlled water source on the unit, the majority of faucet use was to collect warm water for bathing patients. As a solution, the unit was equipped with single-use packages of chlorhexidine wipes in warmers for patient bathing. To prevent accidental use of the hoppers, the faucet handles were removed. Since implementing these changes, prevalence screening has not identified any further cases of CPO carriage or infection over 5 months.

**Conclusion:**

A CPO-KPC outbreak in CVICU was linked to splashback from faucets of hoppers primarily used to collect warm water for sponge-bathing. The outbreak was ended by cessation of faucet use and transition to chlorhexidine wipes for patient bathing.

**Disclosures:**

**All Authors**: No reported disclosures

